# Feasibility and impact of whole-body high-intensity interval training in patients with stable coronary artery disease: a randomised controlled trial

**DOI:** 10.1038/s41598-022-21655-w

**Published:** 2022-10-14

**Authors:** Jacobina Kristiansen, Tórur Sjúrðarson, Erik Lerkevang Grove, Jan Rasmussen, Steen Dalby Kristensen, Anne-Mette Hvas, Magni Mohr

**Affiliations:** 1 Department of Medicine, National Hospital of the Faroe Islands, Tórshavn, Faroe Islands; 2 Department of Clinical Biochemistry, Aarhus University Hospital, Aarhus, Denmark; 3 Department of Cardiology, Aarhus University Hospital, Aarhus, Denmark; 4 Faculty of Health, Aarhus University, Aarhus, Denmark; 5 Faculty of Health, University of the Faroe Islands, Tórshavn, Faroe Islands; 6 Department of Nutrition, Exercise and Sports, University of Copenhagen, Copenhagen, Denmark; 7 Department of Sports Science and Clinical Biomechanics, SDU Sport and Health Sciences Cluster (SHSC), University of Southern Denmark, 5230 Odense M, Denmark

**Keywords:** Cardiology, Quality of life, Randomized controlled trials, Cardiovascular diseases

## Abstract

Exercise training reduces cardiovascular mortality and improves quality of life in CAD patients. We investigated the feasibility and impact of 12 weeks of low-volume high-intensity interval training (HIIT) in CAD-patients. Patients with stable CAD were randomized 1:1 to supervised HIIT or standard care. HIIT sessions were completed three times weekly for 12 weeks on a rowing ergometer. Before and after the 12-week intervention, patients completed a physiological evaluation of cardiorespiratory performance and quality of life questionnaires. Mixed model analysis was used to evaluate differences between and within groups. A total of 142 patients (67 ± 9 years, n_HIIT_ = 64, n_Standard care_ = 78) completed the trial. Training adherence was 97% (range 86–100%). Six patients dropped out because of non-fatal adverse events. Weekly training duration was 54 min with an average power output of 138 W. HIIT increased peak oxygen uptake by 2.5 mL/kg/min (95% CI 2.1–3.0), whereas no change was observed in standard care (0.2 mL/kg/min, 95% CI − 0.2–0.6, P < 0.001). In addition, HIIT improved markers of quality of life, including physical functioning, limitations due to physical illness, general health and vitality (P < 0.05). Twelve weeks of low-volume whole-body HIIT increased cardiorespiratory capacity and improved quality of life in patients with stable CAD compared to standard care. In addition, our study demonstrates that the applied vigorous training regime is feasible for this patient group.

**Clinical trial registration:** www.clinicaltrials.gov. Identification number: NCT04268992.

## Introduction

Ischemic heart disease is a leading cause of mortality worldwide, with 8.9 million annual deaths^1^. Atherosclerosis is the most common cause of myocardial ischemia, and the prevalence of patients with symptomatic coronary artery disease (CAD) is accelerating globally^2^. Thus, feasible and efficient rehabilitation programs, including evidence-based exercise training protocols, are warranted for patients suffering from CAD.

Exercise training is a central component in the treatment of CAD, which is supported at meta-analysis level^3^ and by a large-scale systematic Cochrane review^4^. Current guidelines recommend 30–60 min of moderate-intensity exercise at least five times weekly^5,6^, which may be challenging for frail groups such as CAD patients.

The application of high-intensity interval training (HIIT) has accelerated during the last decade and has been tested in e.g. CAD patients, healthy young individuals and elite athletes^7–10^. Collectively, HIIT protocols in different groups of participants have been demonstrated to upregulate several physiological variables such as maximal oxygen uptake, cardiac function and blood volume as well as skeletal muscle mitochondrial function and angiogenesis^8,11,12^. In recent years, strong clinical interest has arisen in HIIT as an alternative to moderate-intensity continuous exercise^13,14^. Meta-analysis data confirm that HIIT appears to be superior to moderate-intensity continuous training at increasing peak oxygen uptake in patients with CAD, though the effect on quality of life seems to be similar^15–17^. One of the challenges in comparing HIIT protocols from different studies is the relatively large range in total training volume, interval intensities and duration as well as exercise modalities in the various studies being analysed. Furthermore, there is variation in the characteristics of the different CAD populations studied and limited information on the impact and feasibility of HIIT in elderly individuals^18,19^. Thus, further research in the application of HIIT exercise protocols in CAD patients is warranted to specify exercise recommendations that optimise health benefits and reduce risk^20,21^.

The primary objective of the present study was therefore to investigate the effect of low-volume whole-body HIIT on cardiorespiratory performance, physical performance and quality of life in patients with CAD. A secondary aim was to evaluate the feasibility of HIIT for this patient group. We hypothesised firstly that high-intensity rowing has beneficial effects on cardio-respiratory performance, physical performance and quality of life in patients with CAD, and secondly that high-intensity rowing is a feasible exercise protocol for this patient group.

## Methods

### Study design

The study, designed as a randomised controlled trial with two intervention arms, was conducted at the Department of Medicine, National Hospital of the Faroe Islands, Tórshavn, Faroe Islands. Patients were randomly allocated 1:1 to either supervised HIIT or standard care. Patients in the standard care group were asked to continue their lives as usual and did not participate in more than leisure activity. Specifically, they did not change dietary or exercise habits. We obtained written informed consent from all patients, and the Declaration of Helsinki was followed in all respects. The study was approved by the Faroese Ethics Committee and The Faroese Data Inspectorate, and registered at http://www.clinicaltrials.gov (NCT04268992, first registration 13/02/2020). The data that support the results of this study are available upon request from the corresponding author**.**

### Participants

**Figure 1:**
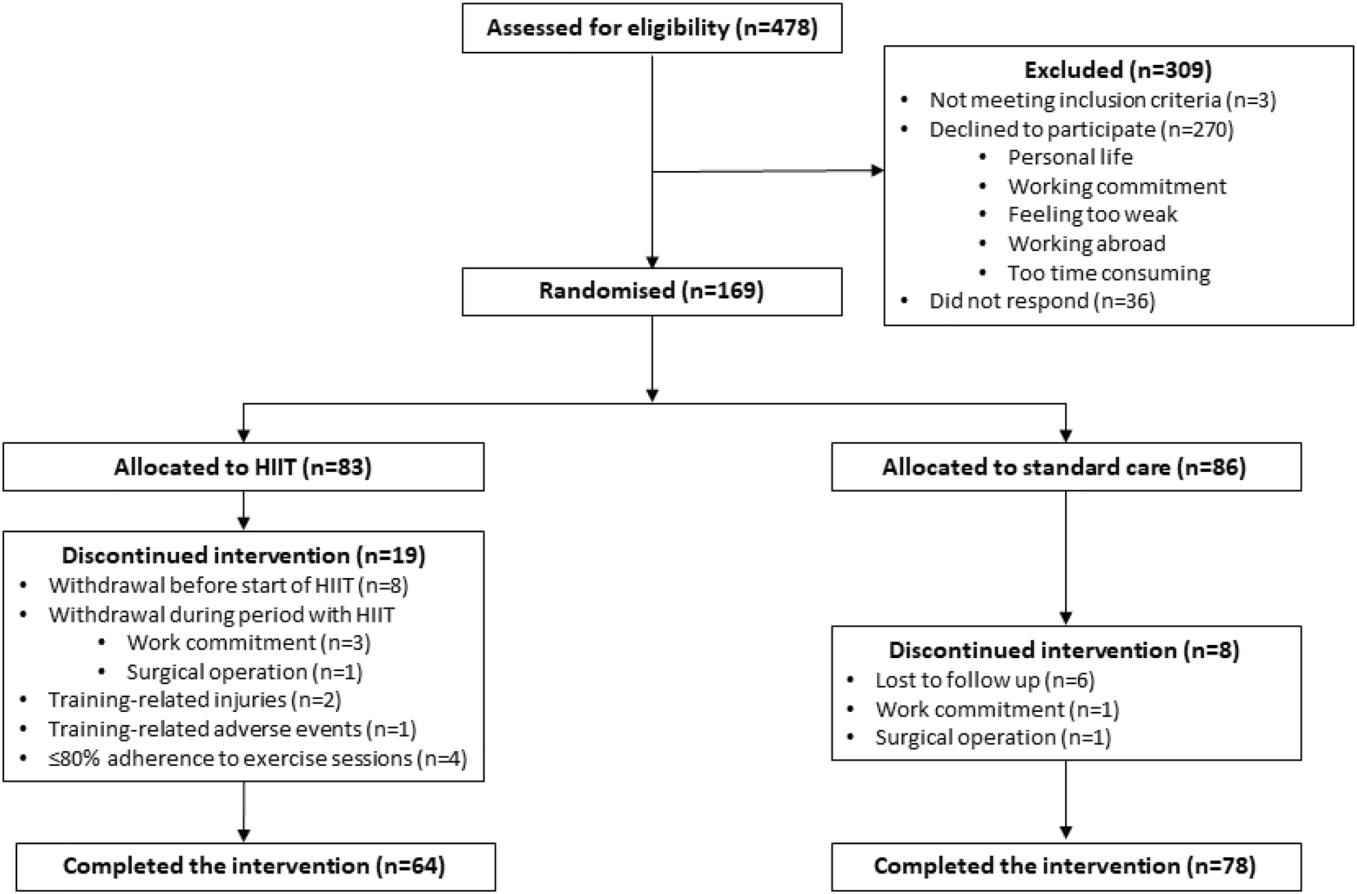
Flowchart of recruitment for the randomised controlled trial. *HIIT* high-intensity interval training.

**Figure 2:**
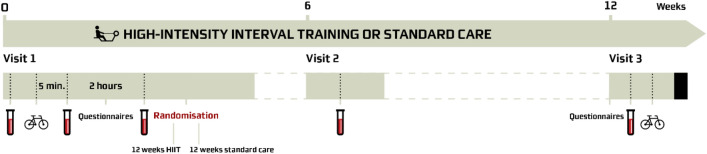
Overview of visits for each patient.

Patients with CAD were identified from discharge summaries from admissions where invasive coronary angiography was performed, and eligible CAD patients received a letter inviting them to participate in the study. The trial flowchart is illustrated in Fig. 1. At baseline, patients were interviewed about their medical history and the following measurements where performed: blood pressure, 12-lead electrocardiogram, blood samples (renal function, lipid profile, haemoglobin A1c), exercise stress test, transthoracic echocardiography (if more than two years since the last scan), body weight and body composition. Furthermore, all patients completed two questionnaires as detailed below. Following the baseline examination, patients were randomised to one of two groups; supervised HIIT or standard care. Each patient visited the hospital at two additional occasions for blood sampling, an exercise stress test and questionnaires (Fig. 2). The same equipment was used before and after the intervention for the exercise stress test and determination of body weight and body composition. The inclusion and the 12-week randomisation period were performed in two rounds: from July to November 2020, and from January to June 2021.

### Inclusion criteria

Patients were included in the study if they were older than 18 years, had angiographically verified CAD treated with percutaneous coronary intervention or coronary artery bypass graft surgery, had previous ST-elevation myocardial infarction/non-ST-elevation myocardial infarction with no need for revascularisation, and at least 12 months since revascularisation or myocardial infarction diagnosis.

### Exclusion criteria

Patients were not invited to participate in the study if they were treated with oral anticoagulants, had severe heart failure (ejection fraction < 30% or New York Heart Association > 2), had been given an implantable cardioverter-defibrillator or undergone cardiac resynchronisation therapy, had severe valvular heart disease, had been hospitalised with serious arrhythmia within the preceding 6 months, or had chronic obstructive pulmonary disease GOLD IV. Moreover, patients were excluded if they were unable to perform strenuous exercise and if they participated in ≤ 80% of the exercise sessions during the intervention period.

### Randomisation

The allocation sequence and randomisation were based on a predetermined block size of 2, 4, 6, and 8 generated by a computerised random number generator in Microsoft Excel (2016). A note with “Exercise” or “Control” was wrapped in aluminium paper and placed in envelopes. An unaffiliated person performed the randomisation and numbered the envelopes from 1 to 169 in a randomised order. Following the completion of all baseline measurements at the initial visit, the patients were given a sealed envelope containing the patient’s study number.

### Exercise test

Patients fasted for ≥ 1.5 h and were instructed to refrain from vigorous exercise for 24 h prior to the experimental testing, and to avoid alcohol, tobacco and caffeine on the day of testing.

Peak oxygen uptake (VO_2peak_) and maximal power output (W_max_) were determined using a modified protocol from a previous study in our research group^22^. The patients completed an incremental cycling test to exhaustion on an electronically braked cycle ergometer (Excalibur Sport, Lode, Groningen, Netherlands) with continuous measurement of VO_2_ using an online gas collection system (model Cosmed, Quark b2, Milano, Italy). Heart rate was monitored (HRM-Dual, Garmin, Olathe, Kansas, USA) throughout the test. Gas analysers and the flow sensor of the applied spirometer were frequently calibrated.

The exercise protocol was initiated with a 6-min warm-up period; 3 min at 30 or 50 W followed by 3 min at 50 or 70 W for women and men, respectively, after which the workload was increased by 15 or 20 W/min for women and men until exhaustion. Patients were verbally encouraged and motivated throughout the test, and they were blinded to pulmonary measurements, power output and time elapsed. Breath-by-breath VO_2_ values and ventilation were averaged over 30 s; the maximal pulmonary ventilation and VO_2peak_ were defined as the highest 30-s value. Submaximal VO_2_ (VO_2submax_) was determined as the average oxygen uptake during the final 30 s of the warm-up interval. Maximal power output was calculated as W_compl_ + 15 (t/60) for women and W_compl_ + 20 (t/60) for men, where W_compl_ is the last fully completed workload and t is the time sustained at the final workload. Maximal heart rate (HR_max_) was defined as the highest measured heart rate, and submaximal heart rate (HR_submax_) was determined as average HR during the final 30 s of the warm-up bout.

### Body composition

Body composition was assessed using bioelectrical impedance analysis under standardised conditions during the laboratory visits (InBody 270, Biospace, California, USA^23^).

### Questionnaires

All patients completed questionnaires about physical activity level (International Physical Activity Questionnaire-Short Form (IPAQ-SF)^24^) and quality of life (Short Form 36 Health Survey Questionnaire (SF-36v2)^25^) at baseline and study end. IPAQ-SF reports physical activity during the last 7 days. Patients in the HIIT group were informed not to include the physical activity related to the intervention in the IPAQ-SF questionnaire. Results in SF-36v2 are presented as T-scores, which are calculated with means and standard deviations from the U.S. general population in 2009 using software from Quality Metric^25^. Dietary habits were not monitored, but patients were instructed to avoid marked changes during the intervention.

### Exercise training

The patients completed ~ 30-min rowing sessions three times weekly for 12 weeks on a wind-braked rowing ergometer with slides (Concept 2 model D w. PM5, Vermont, United States). Ergometer rowing was chosen because it taxes the cardiorespiratory system markedly due to the large active muscle mass^26^. All training sessions were supervised by experienced rowing specialists.

The training program was designed according to the well-documented efficiency of HIIT for improving both cardiovascular and muscular oxidative capacity^8,27^. Training sessions consisted of short-duration (≤ 2 min) exhaustive high-intensity interval bouts utilising a 1:1 work-to-rest ratio. To reduce the risk of injury, the initial 6 sessions of the training intervention were predominantly focused on familiarisation with the rowing ergometer, rowing technique and training modality. Subsequently, an individual target intensity defined as 100% of the average maximum workload (W) from session 7 was provided for each training session. The target intensity was adjusted based on the average maximum workload on session 16 (week 6) and session 25 (week 9) to account for training-induced improvements (see Table S3 in the Supplementary for a detailed overview of the training program).

Power output during the exercise intervals was registered in weeks 3, 6 and 9. The average intensity during the intervals was quantified and normalised to the average power that patients were able to sustain during a 5-min all-out rowing performance test completed in week five. The patients were encouraged to perform as much work as possible during the 5 min and explicitly instructed to pace themselves for a high average power output instead of going all out from the beginning.

### Adverse events

Adverse events were registered continuously and at each hospital visit. Severe adverse events were defined as all-cause mortality, hospitalisation for CAD or atrial and ventricular arrhythmia. An adverse event was considered moderate if it was an exercise injury that caused the patient to withdraw from the study because of musculoskeletal injuries (e.g. back pain, joint pain). Mild adverse events were considered self-limited if it was possible to start and/or restart training (nausea, muscle soreness, fatigue, mild vertigo).

### Statistical analysis

Demographical data are presented as means with standard deviation or medians with 25th and 75th percentiles, and compared using an unpaired t-test or Mann–Whitney test. Proportions are presented in percent and compared using Pearson’s chi squared test or Fisher’s exact test if assumptions were not met for the chi-square test. Changes from baseline to after the intervention are presented as means with 95% confidence intervals [95% CI lower limit; upper limit] unless otherwise stated. Possible between-group differences in continuous endpoints were evaluated for all completed cases by a mixed-model repeated measures approach by means of the SPSS MIXED method^28^. Group (HIIT vs standard care), time (pre vs post intervention) and group × time interaction were specified as fixed factors. The between-group differences in response to the intervention were assessed by the group × time interaction effect. A significant group × time interaction was further evaluated by a Sidak-adjusted pairwise comparison. Random variation and repeated effects were defined from individual patients. Independence of the obtained data was assumed in the model. Visual inspection of homogeneity of residual variance and normality of the residual was performed for all data. If a clear violation of the model assumptions occurred, data were logarithmically transformed to conform to the model assumptions and presented as medians with 95% CI. Finally, possible correlations between changes in VO_2peak_ and markers of quality of life were assessed by the Pearson’s correlation test and an intention to treat analyses was performed on absolute VO_2peak_, VO_2peak_ adjusted for body weight and W_max_. The level of statistical significance was set at P < 0.05. The primary outcomes in the present study were VO_2peak_ and the summary component related to physical health; a marker of quality of life. With an alpha level of 0.05 and a sample size of 64 patients in HIIT and 78 patients in standard care, the trial is provided with 99% and 86% power to detect expected differences in VO_2peak_ and physical component summary, respectively^29^. Sample size was calculated based on markers of fibrinolysis (see clinicaltrials.gov: NCT04268992), and these data will be reported elsewhere. MM has full access to all the data in the study and takes responsibility for its integrity and the data analysis. Statistical analyses were performed using IBM SPSS statistics v.27.0.0.

## Results

### Baseline characteristics

Figure 1 illustrates the recruitment of CAD patients. We sent invitation letters to 478 eligible CAD patients, and 169 agreed to participate in the study. The most common reasons for declining are listed in Fig. 1. In total, 142 patients completed the study; 64 in the exercise group and 78 in the standard care group. Baseline characteristics for the study patients are presented in Table 1. The mean age for all patients was 67 years (33 patients > 75 years), and the majority of the patients (80%) were men. In the standard care group, all patients were treated with acetylsalicylic acid. In the HIIT group, 60 out of 64 patients were treated with acetylsalicylic acid, three patients were treated with clopidogrel and one patient did not take any antithrombotic medication. Treatment with angiotensin-converting enzyme-inhibitors was significantly more common in the HIIT group compared with the standard care group. Moreover, all patients had stable coronary artery disease with a Canadian cardiovascular society-score ≤ 1.

**Table 1:** Patient characteristics.

Characteristics	All (n = 142)	Exercise (n = 64)	Standard care (n = 78)	P-value
Age (years)	66.7 ± 9.4	67.0 ± 9.5	66.4 ± 9.3	0.72
> 75 years	33 (23%)	14 (22%)	19 (24%)	0.82
Gender (male/female)	118/24 (83%/17%)	54/10 (84%/16%)	64/14 (82%/18%)	0.71
BMI, kg/m^2^	29.4 ± 4.8	29.2 ± 4.9	29.7 ± 4.8	0.52
LVEF (%)	57 (56, 58)	57 (55, 58)	57 (56, 59)	0.65
**CAD history**				
Angina pectoris	47 (33%)	22 (34%)	25 (32%)	0.97
NSTEMI	43 (30%)	18 (28%)	25 (32%)	
STEMI	33 (23%)	16 (25%)	17 (22%)	
UAP	18 (13%)	8 (13%)	10 (13%)	
Ischaemic heart failure	1 (1%)	0 (0%)	1 (1%)	
**Treatment**				
PCI	96 (68%)	41 (64%)	55 (71%)	0.47
CABG	49 (35%)	24 (38%)	25 (32%)	0.60
Medical	8 (6%)	3 (5%)	5 (6%)	0.73
**Risk factors and comorbidity**				
Familial predisposition	35 (25%)	19 (30%)	16 (21%)	0.25
Diabetes	27 (19%)	16 (25%)	11 (14%)	0.13
Hypertension	111 (78%)	50 (78%)	61 (78%)	1.00
Dyslipidaemia treatment	135 (95.1%)	61 (95.3%)	74 (94.9%)	1.00
Smoking				
Never smoked	41 (29%)	22 (34%)	19 (24%)	0.38
Ex-smoker	88 (62%)	36 (56%)	52 (67%)	
Current smoker	13 (9%)	6 (9%)	7 (9%)	
Alcohol consumption				
< 7 standard drinks/week	120 (85%)	54 (84%)	66 (85%)	0.14
7–14 standard drinks/week	13 (9%)	4 (6%)	9 (12%)	
> 14 standard drinks/week	8 (6%)	6 (9%)	2 (3%)	
Previous cerebral apoplexy	3 (2%)	2 (3%)	1 (1%)	0.59
Claudicatio intermittens	3 (2%)	2 (3%)	1 (1%)	0.59
Charlson Comorbidity Index	3.8 ± 1.5	4.0 ± 1.8	3.7 ± 1.3	0.27
**Biochemistry**				
Creatinine (µmol/L)	81 ± 21	79 ± 22	82 ± 21	0.44
eGFR (mL/min/1.73m^2^)	79 ± 14	79 ± 15	79 ± 13	0.86
Total cholesterol (mmol/L)	3.6 ± 0.7	3.5 ± 0.6	3.6 ± 0.7	0.24
LDL-C (mmol/L)	1.7 ± 0.5	1.7 ± 0.5	1.8 ± 0.5	0.32
HDL-C (mmol/L)	1.2 ± 0.3	1.2 ± 0.3	1.2 ± 0.4	0.72
Triglycerides (mmol/L)	1.5 ± 1.1	1.4 ± 0.7	1.6 ± 1.3	0.49
HbA1c (mmol/mol)	41 ± 10	43 ± 12	40 ± 8	0.17
**Medications**				
ASA	138 (97%)	60 (94%)	78 (100%)	0.04
Clopidogrel	3 (2%)	3 (5%)	0 (0%)	0.09
Beta blocker	92 (65%)	45 (70%)	47 (60%)	0.22
ACE inhibitor	54 (38%)	31 (48%)	23 (30%)	0.02
Angiotensin II receptor blockers	35 (25%)	12 (19%)	23 (30%)	0.17
Statins	131 (92%)	60 (94%)	71 (91%)	0.75
Ezetimibe	30 (21%)	16 (25%)	14 (18%)	0.41
Ca antagonists	54 (38%)	21 (33%)	33 (42%)	0.30
Nitrates	18 (13%)	8 (13%)	10 (13%)	1.00
Diuretics	31 (22%)	12 (19%)	19 (24%)	0.54

Continuous variables are presented as means ± standard deviations or as medians with 25th and 75th percentiles; dichotomous variables are expressed as numbers and percentages.

*HIIT* high-intensity interval training, *BMI* body mass index, *CAD history* the most severe diagnosis, if the patient has been revascularised multiple times, *LVEF* left ventricular ejection fraction, *CAD* coronary artery disease, *AMI* acute myocardial infarction, *PCI* percutaneous coronary intervention, *CABG* coronary artery bypass graft, *UAP* unstable angina pectoris, *NSTEMI* non-ST-elevation myocardial infarction, *STEMI* ST-elevation myocardial infarction, *eGFR* estimated glomerular filtration rate, *LDL-C* low-density lipoprotein cholesterol, *HDL-C* high-density lipoprotein cholesterol, *HbA1c* Haemoglobin A1c, *ASA* acetylsalicylic acid, *ACE* angiotensin-converting enzyme.

### Exercise training

Each training session started with a 6 min warm-up on the rowing ergometer followed by an average of 12 min of active interval training time. Thus, the total active training time was ~ 18 min/session. The average power output during the warm-up and intervals was 86 ± 34 W and 138 ± 46 W, respectively, corresponding to 72 ± 19% and 117 ± 11% of the average power that the patients could sustain during a 5-min all-out effort. Adherence to the exercise sessions was 97% (86–100%).

### Cardiovascular adaptions

**Figure 3:**
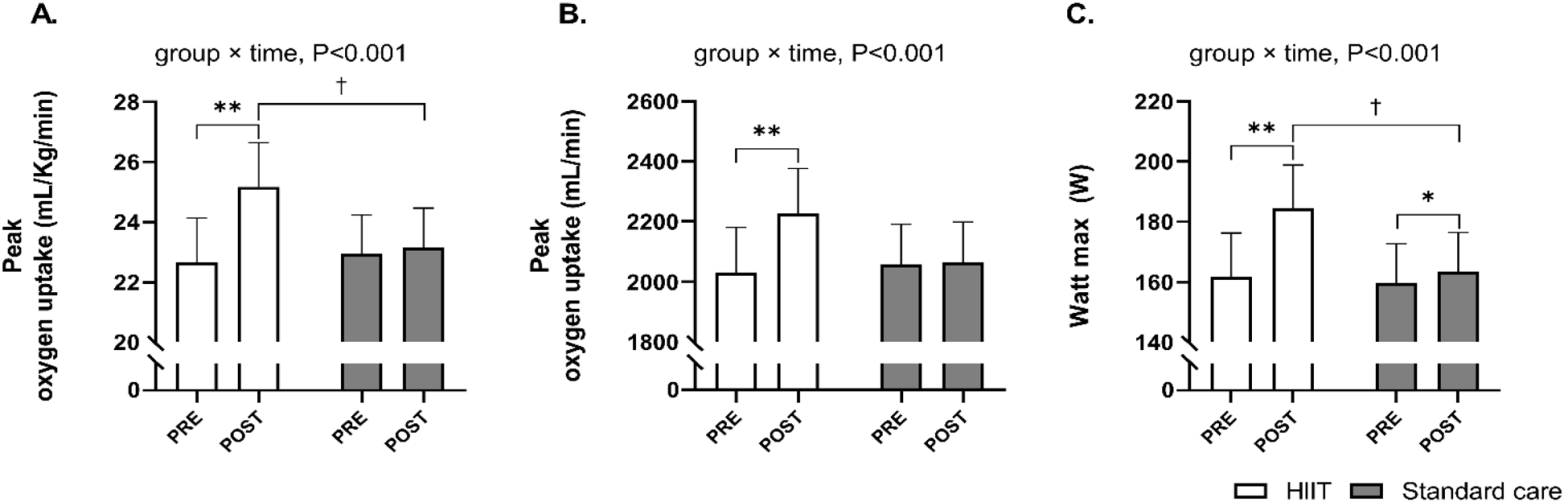
Values are presented as means (with 95% confidence intervals) from a linear mixed-model with group, time, and group × time interaction as fixed factors. The figure shows peak oxygen uptake measured pre- and post-intervention (**A,B**) and peak workload measured pre- and post-intervention (**C**). If a significant effect of group × time interaction existed, the result of the post hoc analysis is indicated by *P < 0.05, **P < 0.001 compared with pre-intervention, and ^†^P < 0.05 compared with standard care.

The applied exercise training regime increased absolute VO_2peak_ and VO_2peak_ adjusted for body weight by 197 mL/min [160; 233] and 2.5 mL/kg/min [2.1; 3.0] (n = 60), respectively, whereas no changes were observed after standard care (8 mL/min [− 24; 46] and 0.2 mL/kg/min [− 0.2; 0.6], group × time interaction P < 0.001; Fig. 3A,B). In contrast, W_max_ improved in both groups, but the improvement was significantly higher in HIIT compared to standard care (23 W [19; 27] (n = 60) vs 3.7 W [0.1; 7.2] (n = 76) group × time interaction P < 0.001; Fig. 3C). The intention to treat analyses on absolute VO_2peak_, VO_2peak_ adjusted for body weight and W_max_ showed similar results compared with the per-protocol analysis. VE_max_ remained constant in standard care (− 0.9 [− 3.1; 1.2] L/min) but increased by 13 L/min [11; 16] in HIIT (group × time interaction P < 0.001; Table 2). In addition, submaximal heart rate decreased by − 5 BPM [− 7;  − 3] in HIIT, while it was unaffected in standard care (− 1.4 BPM [− 3.4; 0.6], group × time interaction P < 0.001; Table 2).

**Table 2:** Cardiovascular adaptions and body composition.

Effect parameter	HIIT			Standard care			Group × time interaction
	Pre	Post	n	Pre	Post	n	
VO_2submax_ (mL/min)	1386 [1335; 1438]	1380 [1329; 1432]	59	1427 [1382; 1472]	1392 [1347; 1437]	76	P = 0.14
HR_submax_ (BPM)	105 [101; 109]	100 [96; 104]*	54	106 [102; 109]	104 [101; 108]	67	P < 0.05
HR_max_ (BPM)	142 [136; 147]	142 [137; 147]	56	141 [136; 146]	140 [135; 145]	68	P = 0.35
VE_max_ (L/min)	83 [77; 89]	96 [91; 102]*^,†^	60	82 [77; 88]	82 [76; 87]	76	P < 0.001
Total body weight (kg)	90 [84; 96]	89 [83; 95]	34	92 [87; 98]	92 [87; 98]	39	P = 0.18
SMM (kg)	36 [34; 38]	36 [34; 38]	34	35 [33; 37]	35 [33; 37]	39	P = 0.20
FM (kg)	26 [22; 30]	24 [21; 28]*	34	30 [27; 34]	30 [26; 33]	39	P < 0.05
%FAT (%)	28 [25; 31]	27 [24; 30]*^,†^	34	32 [29; 35]	32 [29; 34]	39	P < 0.001

Values are presented as means (with 95% confidence intervals) from a linear mixed-model with group, time and group × time interaction as fixed factors.

*HIIT* high-intensity interval training, *VO*_*2submax*_ submaximal oxygen uptake, *HR*_*rest*_ resting heart rate, *HR*_*submax*_ submaximal heart rate, *HR*_*max*_ maximal heart rate, *VE*_*max*_ maximal ventilation, *SMM* skeletal muscle mass, *FM* body fat mass, *%FAT* body fat percentage.

If a significant effect of group × time interaction existed, the result of the post hoc analysis is indicated by *P < 0.001 compared with pre-intervention and ^†^P < 0.05 compared to standard care.

In addition, we performed an exploratory sub-group analysis on patients > 75 years of age. Absolute VO_2peak_ and VO_2peak_ adjusted for body weight increased by 129 mL/min [77; 182] and 1.7 mL/kg/min [1.1; 2.4] in HIIT while it remained unaffected in standard care (− 3 mL/kg/min [− 48; 41] and 0.1 mL/kg/min [− 0.5; 0.6], P < 0.001 for group × time interaction in both analyses).

### Body composition

Exercise training reduced (P < 0.001) body fat mass and body fat percentage by − 1.5 kg [− 2.2;  − 0.8] and − 1.5% [− 2.0;  − 1.0], respectively, whereas no changes were observed in the standard care group (group × time interaction P < 0.05; Table 2). Due to methodological problems, body composition measures were only obtained in half of the patients.

### Quality of life

In general, exercise training increased markers of quality of life associated with physical health which is illustrated by a pronounced improvement (P < 0.001) in physical component summary. Specifically, a group × time interaction (all P-values < 0.05) existed for three out of four components related to physical health; physical functioning, role limitations due to physical illness and general health (Table 3). In addition, a group × time interaction (P < 0.01) existed for one of four components related to mental health; vitality.

**Table 3:** Short form 36 health survey questionnaire, SF-36v2.

Effect parameter	HIIT			Standard care			Group × time interaction
	Pre	Post	n	Pre	Post	n	
Physical functioning	50 [49; 52]	51 [50; 53]*	64	52 [50; 53]	50 [49; 52]*	76	P = 0.001
Role physical	48 [45; 50]	51 [48; 53]	61	48 [46; 51]	48 [46; 51]	76	P < 0.05
Bodily pain	54 [52; 57]	54 [51; 56]	60	51 [49; 53]	50 [47; 52]	76	P = 0.81
General health	50 [48; 52]	52 [50; 54]*	64	51 [49; 52]	48 [47; 50]*	78	P < 0.001
Vitality	54 [51; 56]	57 [55; 59]**^,†^	62	53 [51; 55]	53 [51; 55]	76	P = 0.01
Social functioning	53 [51; 55]	54 [52; 56]	64	53 [52; 55]	53 [52; 55]	77	P = 0.45
Role emotional	50 [48; 52]	52 [50; 54]	60	50 [48; 52]	51 [49; 53]	75	P = 0.43
Mental health	57 [55; 58]	57 [56; 59]	63	54 [53; 56]	55 [54; 57]	76	P = 0.75
Physical component summary	49 [47; 51]	51 [49; 53]*^,†^	60	50 [48; 52]	48 [46; 50]*	73	P < 0.001
Mental component summary	55 [53; 57]	56 [55; 58]	60	54 [52; 55]	55 [53; 56]	75	P = 0.93

Values are presented as a T-score with a mean (with 95% confidence intervals) from a linear mixed-model with group, time and group × time interaction as fixed factors.

*HIIT* high-intensity interval training, *Role-physical* role limitations due to physical problems, *Role-emotional* role limitations due to emotional problems.

If a significant effect of group × time interaction existed, the result of the post hoc analysis is indicated by *P < 0.05, **P < 0.001 compared to baseline and ^†^P < 0.05 compared to standard care.

### Associations between VO_2peak_ and quality of life

The changes in absolute VO_2peak_ were significantly and positively correlated with changes in physical functioning (Pearson’s r = 0.21 [0.04; 0.37], P < 0.05), general health (Pearson’s r = 0.31 [0.15; 0.45], P < 0.001), vitality (Pearson’s r = 0.37 [0.21; 0.51], P < 0.001) and social functioning [Pearson’s r = 0.19 [0.02; 0.34], P < 0.05). Accordingly, a significant correlation existed between changes in VO_2peak_ and changes in both physical component summary (Pearson’s r = 0.24 [0.07; 0.40], P < 0.01) and mental component summary [Pearson’s r = 0.21 [0.04; 0.37], P < 0.05).

### Physical activity

During the 12-week intervention, there was no between-group difference in total physical activity, vigorous activity, moderate activity or walking, nor sitting hours. The results are presented in Supplementary, Table S1. Despite no group × time interaction, it can be noted that patients in the HIIT group demonstrated a significant reduction in walking hours from pre- to post intervention (P < 0.05). Data were log transformed (excluding sitting hours) because they were not normally distributed.

### Lipid profile and haemoglobin A1c

Haemoglobin A1c and lipid parameters did not change over time either between or within groups (Supplementary Table S2).

### Safety and feasibility

One non-fatal severe adverse event was reported due to worsening of angina. Two patients withdrew their consent to participate because of training-related moderate adverse events (lower back pain and knee pain). Mild self-limiting adverse events were reported in three cases (ankle pain, mild vertigo/hypotension, palpitations). In general, the patients gave positive feedback on the exercise training and adherence was high. As expected, the dropout was higher in the HIIT group compared to standard care, 23% vs 9% (P = 0.02), Fig. 1. However, if dropouts before the intervention start are taken into account, the rates were 13% vs 9%, P = 0.33 (adverse event, training-related injuries and adherence ≤ 80%).

## Discussion

In the present study, we randomised CAD patients to either whole-body HIIT or standard care, and demonstrated for the first time that low-volume HIIT training performed on a rowing ergometer, which involves both upper and lower body, was feasible and resulted in significant improvements in VO_2peak_, body fat mass and quality of life. Adherence to the prescribed exercise training sessions was high and the applied HIIT protocol was well tolerated and positively perceived by this patient group with few adverse events and training injuries. There were no fatal adverse events.

### Exercise training and cardiovascular endpoints

We utilized a 12-week interval-based training program consisting predominantly of short-duration (≤ 2 min) high-intensity intervals. The high intensity of the exercise training intervention is confirmed by the average power output of ~ 140 W during the intervals, corresponding to 117% of the average power the patients could sustain during a 5-min all-out rowing test. The efficient training time, including warm-up, totalled only ~ 18 min/session, corresponding to a weekly training volume of ~ 54 min. This is considerably less than the 150 min/week of moderate-intensity aerobic physical activity recommended for patients with CAD by the European Council of Cardiology^6,30^. Despite the low training volume, we observed significantly different changes in VO_2peak_ of 2.2 mL/kg/min between those assigned HIIT compared to standard care. The efficiency of the applied training intervention for upregulating cardiorespiratory fitness is additionally confirmed by the substantial improvements in submaximal heart rate and peak pulmonary ventilation after HIIT only. However, the training-induced changes in VO_2peak_ in the present study are lower compared to a recent meta-analysis of 16 randomised controlled trials^31^, which reported an average VO_2peak_ improvement of 4.52 mL/kg/min in response to 4–12 weeks of exercise training in patients with coronary artery disease. In addition, it was reported that studies with medium-to-long HIIT intervals and studies utilising a work-to-rest ratio > 1 demonstrated the most significant improvements in VO_2peak_^16^. Thus, the application of repetitive exposure to short-duration (≤ 2 min) intervals with a 1:1 work-to-rest ratio, as applied in the present study, may not be optimal for improving cardiorespiratory fitness and may therefore be one of the explanatory factors for the discrepancies between the VO_2peak_ improvements in the present study compared to Du et al.^16^. In contrast, the observed changes in VO_2peak_ in the present study are considerably higher than those reported by a recent meta-analysis^31^ (based on 8 randomised controlled trials) of 436 patients with heart failure with preserved ejection fraction, demonstrating an average VO_2peak_ improvement of 1.7 mL/kg/min in response to 12–24 weeks of exercise-training compared with habitually active controls. In addition, a recent randomised clinical trial amongst 120 patients with heart failure with preserved ejection fraction assigned (1:1:1) to either HIIT (3 × 38 min/week), moderate continuous training (5 × 40 min/week) or guideline control (1 × advice on physical activity) reported numeric changes in VO_2peak_ of 1.1, 1.6 and − 0.6 mL/kg/min, respectively, after 3 months, with no significant difference between HIIT and moderate continuous training^32^. Thus, comparing the training-induced increases in VO_2peak_ after HIIT in Mueller et al.^32^ with the present findings, we observed more than a twofold higher training-induced increase in VO_2peak_ (2.5 vs. 1.1 mL/kg/min) despite a ~ twofold lower weekly training volume (54 vs. 114 min/week), which may indicate a higher susceptibility to exercise training for improving cardiorespiratory fitness in CAD patients compared to heart failure patients.

No a priori-defined minimal clinically difference in VO_2peak_ change was set in the present study. However, previous studies have set it at 2.5 mL/kg/min^32^, which is the exact magnitude of training-induced increase in VO_2peak_ observed after HIIT in the present study, although the between-group difference was marginally lower (2.2 mL/kg/min). Importantly, the level of cardiorespiratory performance is strongly associated with cardiovascular endpoints in healthy individuals as well as patients with cardiovascular disease^33^. Specifically, epidemiological evidence shows that a 1-MET improvement in aerobic capacity is associated with a 13% reduction in all-cause mortality and a 15% reduction in cardiovascular disease events in healthy individuals^34^.

Although Mueller et al.^32^ reported no statistical difference between VO_2peak_ changes in response to HIIT vs. moderate-intensity continuous training in patients with heart failure, accumulating meta-analysis-level evidence shows that HIIT appears to have superior cardiorespiratory benefits compared to moderate-intensity continuous training in patients with CAD^15–18,35,36^. Continuous training is characterised by a constant submaximal workload and steady-state oxygen uptake, whereas HIIT is characterised by short periods of extremely high workload alternated with periods of recovery. The higher efficacy of HIIT is likely caused by the repetitive cardiorespiratory and metabolic strain triggered by the repeated exposure to an intense stimulus, and this is most likely a driver of e.g. cardiovascular remodelling and a resultant increase in aerobic capacity^37^. It is well documented that exercise training plays a central role in the treatment of CAD patients, and current guidelines recommend a comprehensive cardiac rehabilitation program^2,5^. There is evidence supporting beneficial effects of exercise training in patients with CAD in relation to survival rates and a direct mechanistic impact on the pathogenesis is assumed^38–40^. A recent Cochrane meta-analysis review supports this recommendation by demonstrating that long-term exercise training significantly reduces cardiovascular death and hospital admissions in patients with CAD^41^. Accordingly, prior research has shown that CAD patients who continue to be physically active have the lowest mortality risk^42^, however, initiating physical exercise in sedentary and high-risk CAD patients was associated with the greatest reduction in cardiovascular death^43^. However, adhering to time-consuming and high-frequency exercise training program may be challenging for people, such as CAD patients, with a lower general health status and fitness than the general population.

Collectively, the present findings provide compelling evidence of the health-beneficial effects of low-volume HIIT for stable CAD patients compared to standard care, as well as the high feasibility of the applied training modality for this specific patient group.

### HIIT and quality of life

The present study also shows that quality of life improved after the 12 week HIIT intervention compared to standard care. Moreover, there was a significant positive correlation between changes in VO_2peak_ and markers for quality of life, which emphasizes the importance of cardiorespiratory fitness for improving quality of life. However, it is well established that exercise training improves quality of life in CAD patients^41,44^, and recently it was also investigated whether HIIT or moderate-intensity continuous training affected quality of life differently. Indeed, two new meta-analyses demonstrate that both moderate-intensity continuous training and HIIT are equally efficient with regard to upregulated quality of life, even if HIIT has a superior effect on peak VO_2_ gain in patients with CAD^15,16^. In addition to the beneficial effects of the training intervention on quality of life, HIIT also improved variables such as self-rated general health and vitality, which is also supported by other exercise training studies with patient groups^29,45^. These measures are essential for exercise motivation, continuation and adherence to a training program, which indicates that the applied training modality may be sustainable for this type of patient. Future studies should aim to further investigate the psychological effects of HIIT for CAD patients.

### HIIT improves cardiovascular fitness in CAD patients older than 75 years

Patients who participated in our study were older than the patients in the majority of studies examining HIIT in CAD patients^18^ (see Table 1). Previous studies have reported that regular exercise training reduces the risk of cardiovascular disease and mortality in healthy, elderly individuals^46^. Also, a large retrospective study showed that cardiac rehabilitation is associated with decreased mortality in elderly CAD patients (> 65 years of age)^47^, although the underlying mechanisms remain largely unknown. However, a training-induced enhancement in the responsiveness of the β adrenergic receptor, a receptor that deteriorates with aging, may partially explain the improvements in cardiovascular health reported in elderly engaging in regular physical activity^48^.

Several previous reviews call for more evidence on the effect of HIIT compared to moderate-intensity continuous training in elderly with CAD. In particular, the literature calls for data on patients older than 75 years^18,19^. The present study showed that HIIT is safe and improves cardiorespiratory fitness in older CAD patients. Indeed, for this sub-group of CAD patients ≥ 75 years, the HIIT intervention also significantly increased absolute VO_2peak_ and VO_2peak_ adjusted for body weight compared to standard care. Our findings are supported by others using high-intensity interval training in elderly populations^49,50^.

### Effect of HIIT on body composition and low-density lipoprotein cholesterol

Based on body composition assessments, patients in our study were moderately overweight. A training-induced fat loss of 1.5 kg was observed in the HIIT group without loss in total body weight. These findings are supported by previous studies reporting reduced fat mass and increased skeletal muscle mass as well-established consequences of high-intensity training^8,9^, also in the absence of weight loss^51^. Total cholesterol and low-density lipoprotein cholesterol (LDL-C) were not affected by exercise training in this study. In contrast to our findings, Pedersen et al.^52^ reported a reduced LDL-C in response to 12 weeks of aerobic interval training in sedentary overweight patients with CAD. However, these discrepancies may be explained by the observed weight-loss in the exercise group in Pedersen et al.^52^. The body composition data should be interpreted with caution due to the use of bioelectrical impedance technology and not dual-energy X-ray absorptiometry scans being the gold standard.

### Safety

Despite the high intensity of the training, and the whole-body training approach, we only registered two training-related injuries and one adverse event. In conjunction with a training adherence of ~ 97% these findings clearly demonstrate the feasibility of the applied training modality for this elderly and frail patient population.

### Strength and limitations

The strengths of the present study include the systematic and supervised nature of the HIIT intervention, the high compliance, the large sample size and the relatively high average age of the patient population. Moreover, the validity of the cardiorespiratory adaptations should be optimal because the cardiorespiratory test was performed on a cycle ergometer, whilst the exercise intervention was performed on rowing ergometers and thereby minimising the learning effect. Thus, the risk of habituation to the cardiorespiratory test is considered to be minimal. However, some limitations should be considered. The gender ratio was skewed (83% of the patients were males and 17% females) because CAD is more common in men than in than their women counterparts^53^. We cannot exclude that daily medication or comorbidities of the patients may have affected the training response. A total of 65% of the patients received treatment with a beta blocker. Beta blockers lower the heart rate, and may therefore have affected maximal heart rate and other heart rate measures in our study. However, they should not have affected exercise training and cardiorespiratory fitness^54^.

## Conclusion

In conclusion, we demonstrated that 12 weeks of whole-body HIIT improves VO_2peak_ and quality of life in elderly patients with stable CAD. Consequently, a combination of HIIT and standard medical treatment can be advised as a safe, feasible and efficient treatment strategy aimed at improving cardiovascular health and quality of life in patients with stable CAD.

## Supplementary Information

Supplementary Tables.

## Data Availability

The dataset generated and analysed during the current study are available from the corresponding author on reasonable request.

## Abbreviations

CAD: Coronary artery disease
HIIT: High-intensity interval training
VO_2peak_: Peak oxygen uptake
W_max_: Maximal power output
VO_2submax_: Submaximal oxygen uptake
HR_max_: Maximal heart rate
HR_submax_: Submaximal heart rate
IPAQ-SF: International Physical Activity Questionnaire-Short Form
SF-36v2: Short Form 36 Health Survey Questionnaire
