# Supplementary material for: Feasibility and impact of whole-body high-intensity interval training in patients with stable coronary artery disease: a randomised controlled trial
Source: Sci Rep. 2022 Oct 14;12:17295. doi: 10.1038/s41598-022-21655-w (PMC9568554; doi:10.1038/s41598-022-21655-w)
Supplement: Supplementary file 1 — Supplementary Tables. [file 41598_2022_21655_MOESM1_ESM.docx]

**Supplementary**

**Table S1.** International Physical Activity Questionnaire-Short Form (IPAQ-SF).

|  | **HIIT (n=64)** | | | **Standard care (n=78)** | | |  |
| --- | --- | --- | --- | --- | --- | --- | --- |
| **Effect parameter** | **pre** | **post** | **n** | **pre** | **post** | **n** | **group×time interaction** |
| Total | 2132 [1515;3005] | 1742 [1227;2473] | 43 | 1596 [1152;2211] | 1319 [961;1810] | 54 | P=0.97 |
| Vigorous | 1676 [1068;2628] | 1553 [956;2520] | 56 | 1346 [918;1974] | 1043 [692;1573] | 71 | P=0.66 |
| Moderate | 1216 [839;1763] | 967 [679;1376] | 53 | 1057 [733;1522] | 711 [512;984] | 65 | P=0.59 |
| Walking | 893 [669;1193] | 593 [444;792] | 55 | 570 [431;754] | 445 [337;585] | 64 | P=0.50 |
| Sitting (hours) | 6 [5;8] | 6 [5;7] | 53 | 7 [6;8] | 7 [7;8] | 58 | P=0.62 |

HIIT: high-intensity interval training. Total, vigorous, moderate and walking data are presented as physical activity scores in MET-min/week and as medians with 25^th^ and 75^th^ percentiles. Sitting activity is presented in hours with mean and 95% confidence interval. All values are analysed by a linear mixed-model with group, time and group×time interaction as fixed factors.

**Table S2.** Cholesterol and HbA1c levels before and after the intervention.

|  | **HIIT (n=64)** | | | **Standard care (n=78)** | | |  |
| --- | --- | --- | --- | --- | --- | --- | --- |
| **Effect parameter** | **pre** | **post** | **n** | **pre** | **post** | **n** | **group×time interaction** |
| Total cholesterol (mmol/L) | 3.5 [3.3;3.7] | 3.5 [3.3;3.6] | 60 | 3.6 [3.5;3.8] | 3.6 [3.5;3.8] | 75 | P=0.72 |
| LDL-C (mmol/L) | 1.7 [1.6;1.8] | 1.7 [1.5;1.8] | 59 | 1.8 [1.7;1.9] | 1.8 [1.7;1.9] | 72 | P=0.92 |
| HDL-C (mmol/L) | 1.2 [1.1;13] | 1.2 [1.1;1.3] | 60 | 1.2 [1.1;1.3] | 1.2 [1.1;1.3] | 75 | P=0.41 |
| Triglycerides (mmol/L) | 1.4 [1.2;1.7] | 1.3 [1.1;1.6] | 60 | 1.6 [1.4;1.8] | 1.5 [1.3;1.7] | 75 | P=0.76 |
| HbA1c (mmol/mol) | 43 [40;45] | 42 [40;45] | 61 | 40 [38;43] | 40 [38;42] | 78 | P=0.63 |

HIIT: high-intensity interval training. LDL-C: low-density lipoprotein cholesterol. HDL-C: high-density lipoprotein cholesterol. HbA1c: Haemoglobin A1c. Values are presented as means (with 95% confidence intervals) from a linear mixed-model with group, time, and group×time interaction as fixed factors.

**Table S3.** Overview of the applied training program.

| **Week 1** | | | | | | | |
| --- | --- | --- | --- | --- | --- | --- | --- |
| **Session** | **Warm-up (min)*** | **Active interval (min)** | **Rest interval (min)** | **Repetitions** | **Stroke rate (strokes/min)** | **Intensity **** | **Comments** |
| 1 | 06:00 | 01:00 | 01:00 | 10 | Self-selected | - | Focus on familiarisation with the rowing ergometer, rowing technique and training modality |
| 2 | 06:00 | 01:00 | 01:00 | 8 | Self-selected | - |  |
| 3 | 06:00 | 01:30 | 01:30 | 5 | Self-selected | - |  |
|  |  | 3 min rest | | |  |  |  |
|  |  | 00:30 | 00:30 | 5 |  |  |  |
| **Week 2** | | | | | | | |
| 4 | 06:00 | 02:00 | 02:00 | 4 | 22-24 | - | Focus on familiarisation with the rowing ergometer, rowing technique and training modality |
|  |  | 3 min rest | | |  |  |  |
|  |  | 00:30 | 00:30 | 4 | Self-selected | - |  |
| 5 | 06:00 | 01:00 | 01:30 | 8 | Self-selected | - |  |
| 6 | 06:00 | 02:00 | 02:00 | 6 | Self-selected | - |  |
| **Week 3** | | | | | | | |
| 7 | 06:00 | 02:00 | 02:00 | 6 | Self-selected | Max | Intensity perceived as maximum effort |
|  |  | 3 min rest | | |  |  |  |
|  |  | 04:00 |  | 1 | 22-24 | 50% | Cool-down |
| 8 | 06:00 | 01:00 | 01:00 | 7 | Self-selected | 100% |  |
|  |  | 3 min rest | | |  |  |  |
|  |  | 05:00 |  | 1 | 22-24 | 50% | Cool-down |
| 9 | 06:00 | 01:00 | 01:00 | 4 | Self-selected | 100% |  |
|  |  | 2 min rest | | |  |  |  |
|  |  | 00:45 | 00:45 | 4 | Self-selected | 100% |  |
|  |  | 2 min rest | | |  |  |  |
|  |  | 00:30 | 00:30 | 5 | Self-selected | 100% |  |
| **Week 4** | | | | | | | |
| 10 | 06:00 | 02:00 | 02:00 | 7 | Self-selected | 100% |  |
| 11 | 06:00 | 01:00 | 01:00 | 8 | Self-selected | 100% |  |
| 12 | 06:00 | 01:30 | 01:30 | 6 | Self-selected | 100% |  |
|  |  | 3 min rest | | |  |  |  |
|  |  | 00:20 | 00:40 | 6 | Self-selected | 100% |  |
| **Week 5** | | | | | | | |
| 13 | 06:00 | 05:00 |  | 1 | Self-selected | Max | 5-min performance test at an intensity perceived as maximum effort |
|  |  | 4 min rest | | |  |  |  |
|  |  | 10:00 |  | 1 | 22-24 | 50% | Cool-down |
| 14 | 06:00 | 01:30 | 01:30 | 4 | Self-selected | 100% |  |
| 15 | 06:00 | 3 min rest | | |  |  |  |
|  |  | 01:00 | 01:00 | 4 | Self-selected | 100% |  |
|  |  | 3 min rest | | |  |  |  |
|  |  | 00:30 | 00:30 | 4 | Self-selected | 100% |  |
|  |  | 01:30 | 01:30 | 10 | Self-selected | 100% |  |
| **Week 6** | | | | | | | |
| 16 |  | 02:00 | 02:00 | 7 | Self-selected | Max | Intensity perceived as maximum effort |
| 17 |  | 01:30 | 01:30 | 10 | Self-selected | 100% |  |
| 18 |  | 01:00 | 01:00 | 10 | Self-selected | 100% |  |
| **Week 7** | | | | | | | |
| 19 | 06:00 | 01:30 | 01:30 | 8 | Self-selected | 100% |  |
|  |  | 4 min rest | | |  |  |  |
|  |  | 04:00 |  | 1 | 22-24 | 50% | Cool-down |
| 20 | 06:00 | 01:30 | 01:30 | 4 | Self-selected | 100% |  |
|  |  | 4 min rest | | |  |  |  |
|  |  | 01:00 | 01:00 | 6 | Self-selected | 100% |  |
| 21 | 06:00 | 01:00 | 01:00 | 9 | Self-selected | 100% |  |
| **Week 8** | | | | | | | |
| 22 | 06:00 | 01:30 | 01:30 | 8 | Self-selected | 100% |  |
|  |  | 3 min rest | | |  |  |  |
|  |  | 00:30 | 00:30 | 4 | Self-selected | 100% |  |
| 23 | 06:00 | 02:00 | 02:00 | 6 | Self-selected | 100% |  |
| 24 | 06:00 | 02:00 | 02:00 | 8 | Self-selected | 100% |  |
| **Week 9** | | | | | | | |
| 25 | 06:00 | 02:00 | 02:00 | 7 | Self-selected | Max | Intensity perceived as maximum effort |
| 26 | 06:00 | 01:30 | 01:30 | 10 | Self-selected | 100% |  |
| 27 | 06:00 | 01:00 | 01:00 | 10 | Self-selected | 100% |  |
| **Week 10** | | | | | | | |
| 28 | 06:00 | 02:00 | 02:00 | 8 | 22-24 | 100% |  |
| 29 | 06:00 | 01:00 | 01:00 | 8 | Self-selected | 100% |  |
|  |  | 3 min rest | | |  |  |  |
|  |  | 05:00 |  | 1 | 22-24 | 50% | Cool-down |
| 30 | 06:00 | 01:30 | 01:30 | 2 | Self-selected | 100% |  |
|  |  | 3 min rest | | |  |  |  |
|  |  | 01:00 | 01:00 | 3 | Self-selected | 100% |  |
|  |  | 3 min rest | | |  |  |  |
|  |  | 00:45 | 00:45 | 4 | Self-selected | 100% |  |
|  |  | 3 min rest | | |  |  |  |
|  |  | 00:30 | 00:30 | 5 | Self-selected | 100% |  |
| **Week 11** | | | | | | | |
| 31 | 06:00 | 02:00 | 02:00 | 7 | Self-selected | 100% |  |
| 32 | 06:00 | 01:30 | 01:30 | 8 | Self-selected | 100% |  |
| 33 | 06:00 | 01:00 | 01:00 | 12 | Self-selected | 100% |  |
| **Week 12** | | | | | | | |
| 34 | 06:00 | 00:45 | 01:00 | 8 | Self-selected | 100% |  |
|  |  | 4 min rest | | |  |  |  |
|  |  | 04:00 |  | 1 | 22-24 | 50% | Cool-down |
| 35 | 06:00 | 02:00 | 02:00 | 6 | Self-selected | 100% |  |
| 36 | 06:00 | 05:00 |  | 1 | Self-selected | Max | Intensity perceived as maximum effort |
|  |  | 4 min rest | | |  |  |  |
|  |  | 04:00 |  | 1 | 22-24 | 50% | Cool-down |

*The standardised 6-min warm-up was initiated with a 2 min low-intensity rowing, and after that, 10 all-out effort strokes were performed at the start of each minute (i.e. 3^rd^, 4^th^, 5^th^ and 6^th^ min), followed by low to moderate-intensity rowing for the remainder of the minute. **The individualized target intensity was 100% of the average maximal workload (W) from session 7. The target intensity was adjusted based on the average maximal workload (W) on session 16 (week 6) and session 25 (week 9) to account for training-induced improvements.
